# Problem Gambling in Early Adulthood: a Population-Based Study

**DOI:** 10.1007/s11469-020-00401-1

**Published:** 2020-10-26

**Authors:** Alan Emond, Mark D. Griffiths, Linda Hollén

**Affiliations:** 1 Centre for Academic Child Health, University of Bristol Medical School, Bristol, UK; 2 International Gaming Research Unit, Psychology Department, Nottingham Trent University, Nottingham, UK

**Keywords:** Gambling, At-risk gambling, Problem gambling, Youth, Young adults, ALSPAC

## Abstract

The aims of this study were to investigate stability of problem gambling between 20 and 24 years of age, and the antecedents and consequences of problem gambling at age 20 years. Young adult participants in the Avon Longitudinal Study of Parents and Children (ALSPAC) completed computer-administered gambling surveys on paper, or online. Responses to the Problem Gambling Severity Index (PGSI) were complete for 2624 participants at 20 years, and 1921 participants at 24 years. Responses were categorized into ‘non-problem’ (71–78%), ‘low-risk gambling’ (16–21%), ‘moderate-risk gambling’ (4–5.5%), and ‘problem gambling’ (1–1.5%). The overall frequency of moderate-risk/problem gambling varied little between age 20 and 24 years, and scratch cards, online betting and gambling were the most frequent activities. Problem gamblers at age 20 years had a history of hyperactivity and conduct problems in adolescence, high sensation seeking, and an external locus of control. They were more likely to have mothers who had problems with gambling, reported less parental supervision, and higher social media usage. Moderate-risk/problem gambling at age 20 years was associated with regular cigarette smoking, high levels of illicit drug use, and problematic use of alcohol at age 24 years. A significant minority of young adults (mainly males) showed problem gambling behaviours which appeared to be established by the age of 20 years and were associated with other potentially addictive behaviours.

**Electronic supplementary material:**

The online version of this article (10.1007/s11469-020-00401-1) contains supplementary material, which is available to authorized users.

Young people, defined here as being 15–24 years of age, are known to be at risk of problems with gambling because of cognitive immaturities and lack of development of executive function, which increase impulsivity and risk-taking behaviours (Chambers and Potenza 2003). In the UK (where the present study was carried out), this vulnerability may increase given the expanding opportunities for young people to gamble through online gaming, fixed odds terminals, and in-play betting (Griffiths and Parke 2010). The UK Gambling Commission’s recent report into adolescent gambling (Gambling Commission 2019) showed that although rates of gambling under 16 years had fallen from 23% in 2012 to 11% in 2018, there had been an increase in online activity and the buying of loot boxes in video gaming. The 2018 Health Survey for England reported that 45% of men and 33% of women aged 16–24 years reported gambling in the past year, and 20% of men and 2% of women gambled online (NHS Digital 2019).

A recent systematic review on adolescent gambling (Calado et al. 2017) concluded that a small but significant minority (range 0.2–12%) of adolescents have gambling-related problems. The adverse consequences of problematic gambling for young people include negative emotional states, poor educational and vocational outcomes, and difficulties in family or peer relationships (Hardoon et al. 2004). Most of this evidence comes from cross-sectional study designs, with few prospective studies of long-term consequences in late adolescence and young adulthood. Longitudinal studies in Canada (Vitaro et al. 2001), Australia (Delfabbro et al. 2014,), and the USA (Bray et al. 2014) which have followed adolescents across the transition to adulthood report that rates of gambling increase gradually with age, increasing for some activities (e.g. online betting) while decreasing for others (e.g. card games). Problem gambling may rise or decline in young adulthood, but long-term problem gamblers experience the greatest number of poor behavioural outcomes (Scholes-Balog et al. 2016). Although prior gambling is predictive of subsequent behaviour, there is considerable within-person inconsistency, and individuals change preferences for different types of games (Delfabbro et al. 2014).

A systematic review of the early risk and protective factors for problem gambling by Dowling et al. (2017) organized factors according to a socio-ecological model into individual, relationship, community, and society levels. The individual developmental antecedents of problem gambling among young people include male gender, impulsivity, sensation seeking, depression, and anti-social or violent behaviour. Temperament observed as early as 3 years old (Slutske et al. 2005,), and higher temperamental frustration in adolescence (Yücel et al. 2015) are predictive of problem gambling in early adulthood. Depression has been reported to be both an antecedent (Dussault et al. 2011) and a consequence of problem gambling (Chinneck et al. 2016). Relationship influences on gambling problems in young adulthood include parental gambling (Winters et al. 2002), low levels of parental monitoring in adolescence (Lee et al. 2014), and anti-social behaviour among peers (Scholes-Balog et al. 2016). Poor academic performance at school or college is an important community level influence (Fröberg et al. 2015) and can be both antecedent and a consequence of problem gambling (Griffiths 2002).

With the recognition that young people are especially vulnerable to problems with gambling, and the evidence that online gambling appears to be increasing among the 16–24-year age group in the UK, more information is needed about how problem gambling evolves in young people so that gambling-related harm can be prevented. The Avon Longitudinal Study of Parents and Children (ALSPAC), a population-based cohort study in south-west England, has provided an opportunity to prospectively examine the frequency, antecedents, and consequences of gambling in the 17–24-year age group. We have previously published that any gambling in the past year was reported by 54% of 17-year-olds, rising to 68% at 20 years, and 66% at 24 years (Hollen et al. 2020). There was with a heavy male bias in those participants admitting regular weekly gambling, and rates of regular gambling were stable at 11–12% between age 20 and 24 years. The most common forms of gambling were playing scratch cards, playing the lottery, and private betting with friends, although betting and gambling via online sources increased markedly at 20 and 24 years compared with 17 years.

We now report on problem gambling in this cohort, investigating the stability of problem gambling from age 20 to 24 years; antecedents of problem gambling; and the mental health, substance abuse, and social consequences at age 24 of problem gambling at age 20. It was hypothesised that (i) rates of problem gambling would be stable between 20 and 24 years, (ii) problem gambling would be more common in young people with a history of parental gambling and individual developmental traits such as hyperactivity and sensation seeking, and (iii) problem gambling would be associated with increased rates of depression and other potentially addictive behaviours at age 24 years.

## Methods

### ALSPAC Cohort

The Avon Longitudinal Study of Parents and Children (www.bristol.ac.uk/alspac) is a birth cohort study which enrolled mothers in early pregnancy in the Bristol and surrounding areas in England in 1991–1992. It has detailed information on parents (Fraser et al. 2012) and children (Boyd et al. 2013), collected prospectively at multiple times during pregnancy and throughout childhood. Sources of data include self-report surveys; clinical assessments; birth, medical, and educational records; and biological samples. The initial recruitment resulted in a core cohort of 14,541 pregnancies and 13,988 children alive at 12 months. A total of 913 additional participants have been enrolled in the study since the age of 7 years with 195 of these joining since the age of 18 years. This additional enrolment provides a baseline sample of 14,901 participants who were alive at 1 year of age.

The study website contains details of all the data that are available through a fully searchable data dictionary (http://www.bris.ac.uk/alspac/researchers/data-access/data-dictionary/). Ethical approval for the ALSPAC was obtained from local research ethics committees. Informed consent for the use of data collected via questionnaires and clinics was obtained from participants following the recommendations of the ALSPAC Ethics and Law Committee at the time.

### Gambling Participants

A total of 9000 young participants in the ALSPAC were invited to complete gambling surveys either on paper or online as part of a bigger survey on other aspects of the participants’ life, when the young people were aged 20 years (2012–2013) and 24 years (2016–2017). If a participant completed a paper survey, the data were entered by ALSPAC staff into online databases.

### Gambling Activity

Young people’s participation in specific gambling activities during the past year was assessed at both time points using items derived from the British Gambling Prevalence Survey 2007 (Wardle et al. 2008) (see Supplementary Table 1). Problems with gambling were assessed using the Problem Gambling Severity Index (PGSI), a nine-item scale derived from a 31-item larger screen, the Canadian Problem Gambling Inventory (Ferris and Wynne 2001). Only participants reporting gambling in the past year were asked to complete the PGSI, and respondents were categorized as non-problem gamblers (score of 0), low-risk gamblers with few or no identified negative consequences (score 1 or 2), moderate-risk gamblers leading to some negative consequences (score 3 to 7), and problem gamblers with negative consequences and possible loss of control (score 8 or more). Due to low numbers, moderate-risk gamblers were pooled with problem gamblers for analyses, as has been done in many previous studies (Canale et al. 2016; Potenza et al. 2011). The term ‘at-risk’ gambling includes both the low-risk and the moderate-risk/problem gambling groups. The gambling activity of the mothers was captured separately using the PGSI reported in a self-completion questionnaire when the children were aged 18 years in 2010–2011.

### Antecedents of Problem Gambling

The choice of antecedents was informed by previous research by the study team and reviews of the gambling literature (Griffiths 2011), and were clustered into child, parental, and socio-economic (SES) factors. Child variables included gender, IQ measured in a research clinic at age 8 years using a short version of the WISC III (lowest quartile, IQ < 90), frequency of video gaming reported by questionnaire at age 13–14 years, smoking and alcohol usage reported by questionnaire at 16.5 years, hyperactivity and conduct problems reported at age 16.5 years using the Strengths and Difficulties Questionnaire, locus of control assessed at age 16.5 years, sensation seeking at age 17 years, education/employment status at age 17, diagnosed depression at age 17 years, and mental well-being at 17.5 using the Warwick-Edinburgh Mental Well-being Scale (WEMWBS). Parental and SES variables included maternal highest education level in pregnancy, financial difficulties in pregnancy, maternal problem gambling when child was aged 18 years, and parental monitoring (reported by young people aged 17 years—see Supplementary Table 2). More detailed information about the variables used is provided in Supplementary Table 3.

### Outcomes of Problem Gambling

Outcomes used for investigating effects of problem gambling in young adulthood were depression, anxiety, self-harm, criminal activity, use of illicit drugs, smoking cigarettes, use of alcohol, and employment. All outcomes were collected by self-report using online and paper questionnaires when participants were aged 24 years. More detailed information about these outcome variables is provided in Supplementary Table 3.

### Analytic Plan and Statistical Methods

All available data were used to conduct univariate analyses on the stability of problem gambling and the antecedents to problem gambling. Categorical data were analysed using Chi-square tests and numerical data using ANOVA. For categorical variables, we also looked at adjusted residuals after the Chi-square tests to see if the observed cell counts for the reference category differed to that expected. The larger the residual, the more different from the average values and that expected. A general rule of a residual of 2 was used as signifying a large enough difference (equating to 95% confidence).

Variables significant at *p* < 0.10 in the univariate analyses were then included as covariates in multivariate analyses. Due to the number of antecedents analysed, a large loss to follow-up and missing data on antecedents, outcomes, and the PGSI, multivariate analyses adjusting for other significant factors would not be possible without multiple imputation. Multiple imputations using 50 imputations by the chained equations method were performed utilizing the mi impute command in STATA v.15. Variables included in the imputation model were all of those included in the final regression models (many of which predicted missingness in the other variables). Additionally, a few auxiliary variables also associated with missingness (stressful live events score, maternal age at birth, gambling regularity at age 17 and 24 years; see Supplementary Material 1 for more details). We included non-gamblers in the analyses, so imputed up to the number of participants where gambling status (yes/no) at age 20 years could be established (*N* = 4263). PGSI was imputed conditional on gambling status (i.e. only those who did gamble were imputed for this variable). A comparison of the unadjusted associations using all available data and the imputed dataset is contained in Supplementary Table 4. Adjustment for other variables was carried out in a stepwise procedure using binomial (for binary variables) or multinomial (for variables with > 2 categories) logistic regressions: (i) adjusted for all child variables; (ii) adjusted for child variables and parental variables; and (iii) adjusted for child variables, parental variables, and SES variables. A final minimal model was created adjusting only for variables where confidence intervals did not cross 1 to avoid problems from over-adjusting. Unadjusted and minimal adjusted models (fully adjusted for significant covariates) are shown in main results, and models adjusted for child variables and family variables separately can be provided on request. Odds ratios and 95% confidence intervals (95% CI) are reported.

## Results

### Frequency and Stability of Problem Gambling

**Fig. 1:**
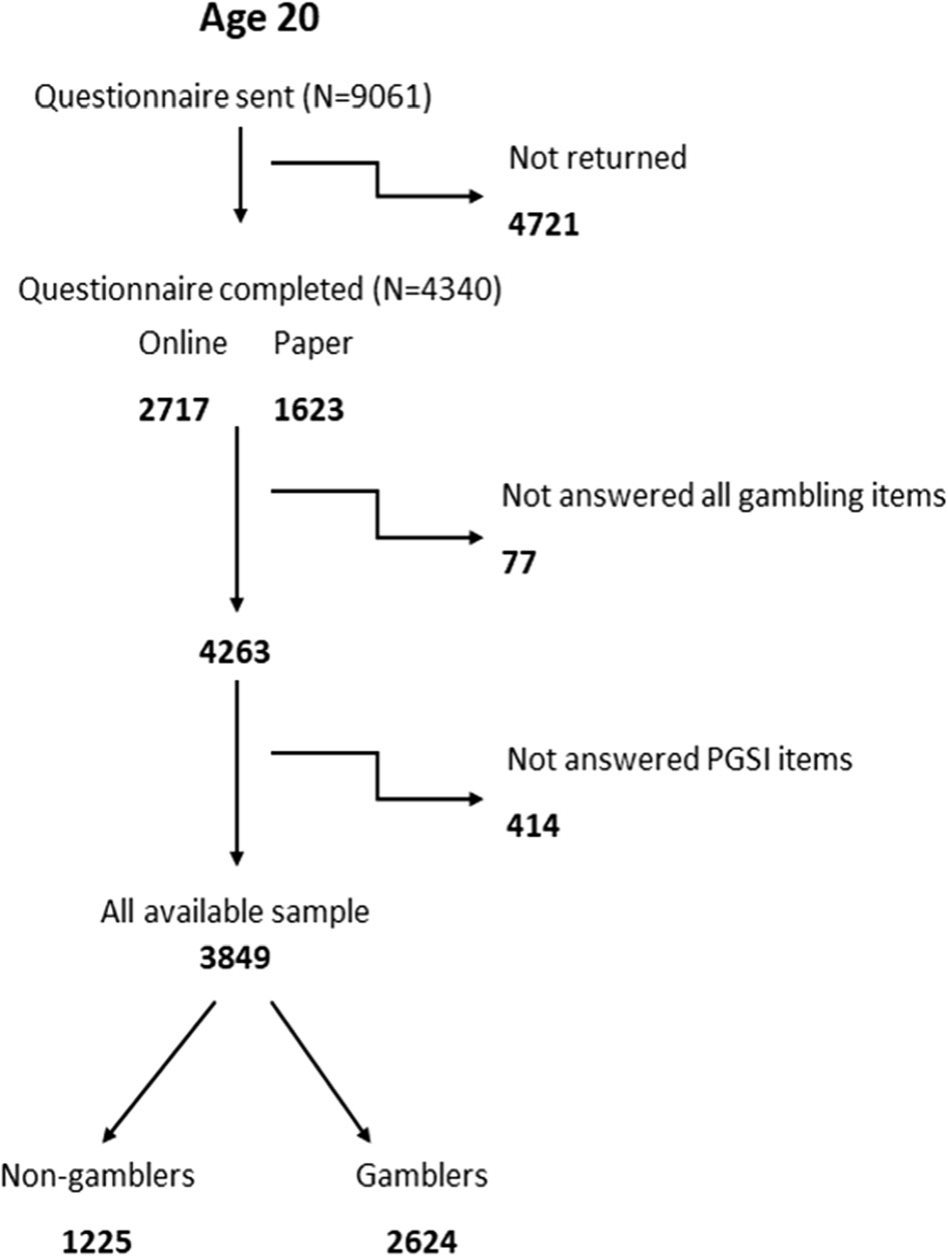
Flow chart showing all available data for participants aged 20 years

**Fig. 2:**
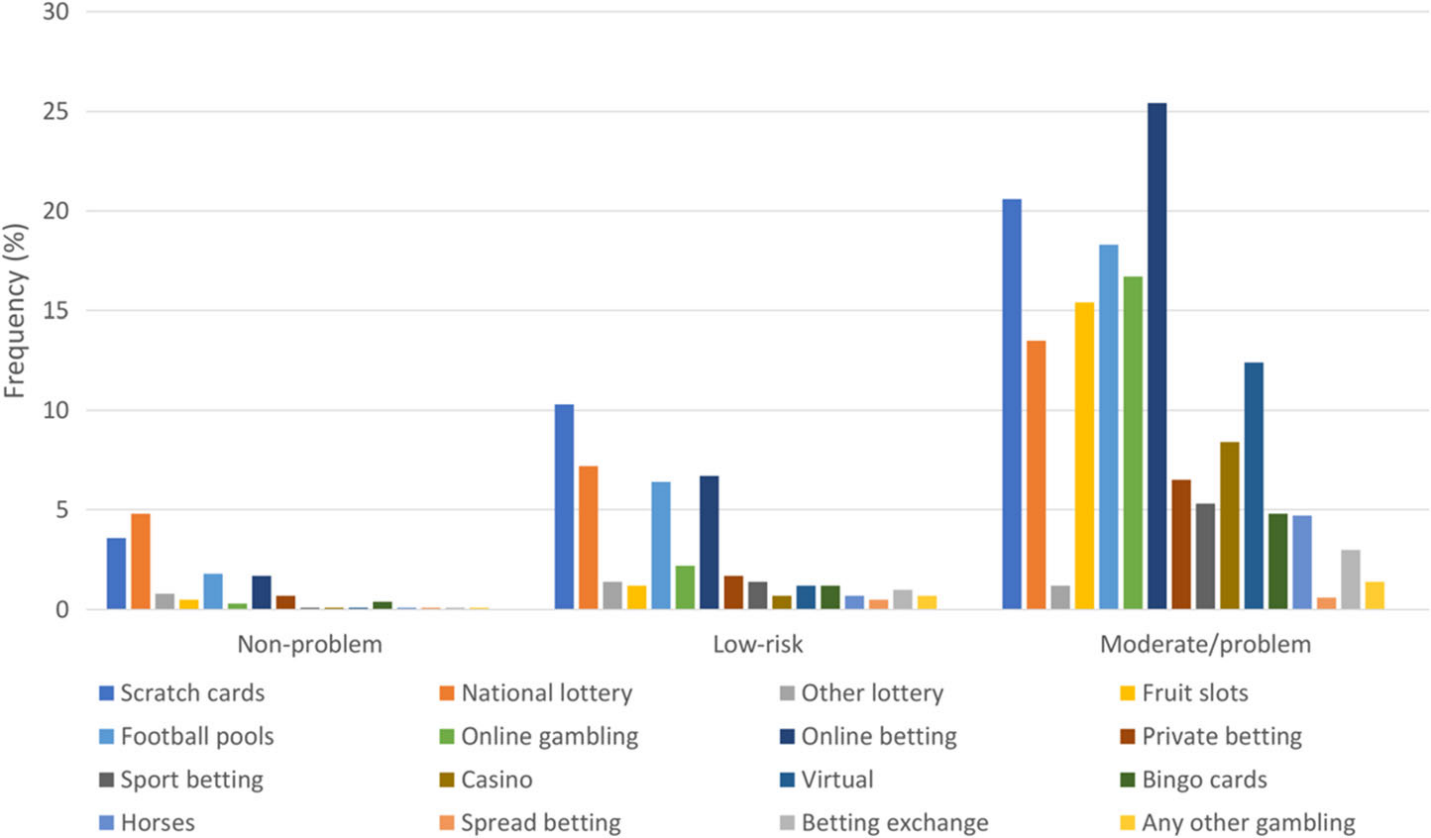
Distribution of gambling activities in regular gamblers (> = weekly), categorized into non-problem, low-risk, and moderate-risk/problem gamblers

**Fig. 3:**
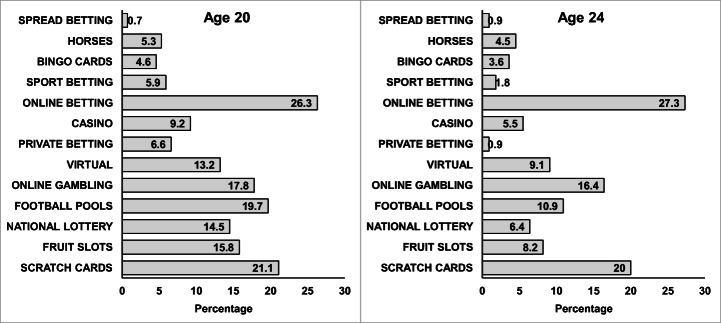
Distribution of gambling activities at 20 and 24 for moderate/problem gamblers who gambled every week

Figure 1 shows participant flow at age 20 years. Out of 9061 questionnaires sent out, 4263 (48%) were returned of which 2624 participants answered all PGSI items. More females (61%) than males completed the PGSI. The distribution of gambling activities among non-problem gamblers, low-risk gamblers, and moderate-risk/problem gamblers for those that gambled regularly is illustrated in Fig. 2. This shows that moderate-risk/problem gamblers gambled on a wide range of activities, particularly on the internet, whereas non-problem gamblers limited their gambling to the lottery, scratch cards and football pools, and playing bingo. Figure 3 shows the change in distribution of activities between age 20 and 24 years for moderate-risk/problem regular gamblers only. Similar to age 20 years, at 24 years moderate-risk/problem gamblers gambled on a wide variety of activities, with no activity increasing in frequency, but some activities like sport betting, football pools, and private betting decreased in frequency. The amount spent/month on gambling at age 24 years showed a clear trend, with 92% of non-problem gamblers spending < £10/month, whereas in the group of moderate-risk gamblers 29% spent < £10 and 56% spent £11–£100/month. Problem gamblers spent the most, with 24% reporting spending £11–£100, and 62% spending > £100/month.

The proportion of participants in the combined moderate-risk/problem gambling group remained stable across time from 6.5% at 20 years to 6.9% at 24 years, whereas the proportions of low-risk gamblers decreased, and non-problem gamblers increased from age 20 to 24 years (Table 1). There was a strong male gender bias: males comprised 74.1% of the moderate-risk/problem group at 20 years, and 67% at 24 years. Moderate-risk/problem gambling was strongly associated with regular (at least weekly) gambling at both 20 and 24 years. Analysis of the 995 participants (38%) who provided PGSI data at 20 and at 24 years (Table 2) clearly shows that being a moderate-risk/problem gambler at age 20 years was highly predictive of being one at age 24 years (49% compared with 8% of low-risk gamblers and 1% of non-problem gamblers at age 20 years).

**Table 1:** Proportion of participants in each PGSI category (all available data)

	Non-problem gamblers (score 0)	Low-risk gamblers (score 1–2)	Moderate-risk gamblers (score 3–7)	Problem gamblers (score > =8)	*N*
20 years	1866 (71.1%)	588 (22.4%)	145 (5.5%)	25 (1.0%)	2624
24 years	1503 (78.2%)	305 (15.9%)	84 (4.4%)	29 (1.5%)	1921
*p* value	< 0.001	< 0.001	0.08	0.90

**Table 2:** Transition between gambling states from age 20 to age 24 years (all available data)

*N* = 995	Age 24 years		
Age 20 years	Non-problem	Low risk	Moderate/problem
Non-problem			
Observed *N*	610	74	7
Observed %	88.28	10.71	1.01
Adjusted residual	*12.29*	− 7.62	− 9.78
Low risk			
Observed *N*	141	77	18
Observed %	59.75	32.63	7.63
Adjusted residual	− 7.47	*7.52*	1.35
Moderate/problem			
Observed *N*	20	15	33
Observed %	29.41	22.06	48.53
Adjusted residual	− 9.83	1.23	*15.57*

### Antecedents of Moderate-Risk/Problem Gambling

Univariate analyses of demographic factors associated with moderate-risk/problem gambling at age 20 years using all available data are presented in Table 3. Important individual factors associated with subsequent moderate-risk/problem gambling were male gender, preference for video gaming at 13 years, higher scores on hyperactivity and conduct difficulties measured at 16 years, an external locus of control measured at 16 years, high sensation seeking scores at 17 years, and weekly smoking and alcohol use at 16 years. However, locus of control proportions and means of sensation seeking scores did not differ greatly between low-risk and moderate-risk/problem gambling groups. For male participation and preference for video gaming, adjusted residuals showed that observed frequencies were higher than expected for both low-risk and moderate-risk/problem gambling. The proportion of regular smokers and alcohol users also increased with increased severity of problem gambling, but smoking proportions did not differ between low-risk and moderate-risk/problem gamblers. Family characteristics which were risk factors for moderate-risk/problem gambling were financial difficulties, lower maternal education level, a history of maternal problem gambling, and lower levels of parental monitoring reported by young people at 17 years.

**Table 3:** Univariate associations between demographic covariates and PGSI at age 20 years (all available data)

Covariates	Non-gamblers (*N*_tot_ = 1225)	Non-problem (*N*_tot_ = 1868)	Low risk (*N*_tot_ = 586)	Moderate risk/problem (*N*_tot_ = 170)	*p* value
Individual factors					
Gender (% male)	32.1%	38.3%	51.9%	74.1%	< 0.001
Adjusted residuals	*− 6.84*	*− 2.10*	*6.47*	*9.29*	
IQ at 8 years (% lowest quartile)	14.9% *n* = 967	15.9% *n* = 1464	20.2% *n* = 444	18.5% *n* = 135	0.07
Adjusted residuals	− 1.48	−0.62	*2.42*	0.70	
Playing video games at 13 years (% yes)	53.7% *n* = 940	59.4% *n* = 1401	65.8% *n* = 398	81.6% *n* = 125	< 0.001
Adjusted residuals	*− 4.32*	− 0.01	*2.82*	*5.17*	
Hyperactivity at 16.5 years (% abnormal; score 7–10)	3.2% *n* = 964	3.3% *n* = 1407	5.1% *n* = 395	10.3% *n* = 116	0.001
Adjusted residuals	− 1.13	− 1.41	1.44	*3.78*	
Conduct problems at 16.5 years (% abnormal; score 4–10)	3.7% *n* = 962	3.3% *n* = 1406	4.1% *n* = 394	9.5% *n* = 116	0.01
Adjusted residuals	− 0.16	− 1.31	0.27	*3.25*	
Locus of control at 16.5 years (% > median [external])	34.2% *n* = 951	33.8% *n* = 1339	42.1% *n* = 397	45.0% *n* = 109	0.003
Adjusted residuals	− 1.04	− 1.86	*2.91*	*2.08*	
Sensation seeking at 17 years (mean [SD])	51.0 (7.3) *n* = 778	52.3 (7.2) *n* = 1071	53.6 (7.4) *n* = 301	54.6 (5.9) *n* = 65	< 0.001
Depression at 17 (ICD-10 diagnosis yes)	8.7% *n* = 838	6.6% *n* = 1223	9.0% *n* = 354	12.4% *n* = 97	0.08
Adjusted residuals	1.09	*− 2.28*	0.87	1.67	
Mental well-being at 17.5 years (% lowest quartile)	29.5% *n* = 845	24.7% *n* = 1248	29.7% *n* = 350	33.7% *n* = 92	0.03
Adjusted residuals	1.73	*− 2.91*	1.09	1.40	
Education/employment at 17 years (% not in education/employment)	8.8% *n* = 1176	7.2% *n* = 1797	7.2% *n* = 559	12.4% *n* = 161	0.06
Adjusted residuals	*− 3.40*	0.67	*3.00*	1.17	
Cigarette smoking at 16.5 years (weekly)	14.1% *n* = 1197	19.2% *n* = 1826	23.2% *n* = 573	29.4% *n* = 160	< 0.001
Adjusted residuals	*− 2.72*	1.42	1.11	1.02	
Alcohol use at 16.5 years (% weekly)	7.8% *n* = 1084	10.8% *n* = 1735	18.3% *n* = 540	34.0% *n* = 162	< 0.001
Adjusted residuals	*− 2.01*	0.34	*2.16*	1.79	
Family factors					
Maternal education 32 years w/gest. (% with > A level)	29.0% *n* = 1150	18.3% *n* = 1752	13.8% *n* = 528	12.6% *n* = 159	< 0.001
Adjusted residuals	*8.25*	*− 3.59*	*− 4.28*	*− 2.62*	
Financial difficulties 32 years w/gest. (% difficulty score > 3)	21.6% *n* = 1120	25.3% *n* = 1715	26.1% *n* = 517	33.3% *n* = 156	0.008
Adjusted residuals	*− 2.79*	0.91	0.88	*2.60*	
Maternal problem gambling child age 18 years (% at-risk/problem)	1.0% *n* = 790	2.3% *n* = 1111	2.0% *n* = 297	7.7% *n* = 91	< 0.001
Adjusted residuals	*− 2.55*	0.94	− 0.04	*3.87*	
Parental monitoring at 17 years (% lowest quartile)	21.7% *n* = 824	24.1% *n* = 1152	30.8% *n* = 344	33.6% *n* = 110	0.001
Adjusted residuals	*− 2.43*	− 0.61	*2.84*	*2.23*	

### Outcomes of Moderate-Risk/Problem Gambling

The frequency of participants in the different categories of PGSI remained very similar in the imputed dataset compared with all available data (Table 4). Unadjusted and adjusted multivariate analysis of moderate-risk/problem gambling at 20 years against outcomes collected at 24 years are summarized in Table 5 for all six pairwise PGSI category comparisons using the imputed dataset. Depression showed an association with any at-risk gambling, in that both low-risk and moderate-risk/problem gamblers were more likely to be depressed at 24 years compared with non-problem gamblers. Adjustment had very little effect on depression outcomes. However, those that did not gamble at all were more anxious and depressed than non-problem gamblers, suggesting a less straightforward relationship between gambling and anxiety/depression. Regular cigarette smoking and intake of illicit drugs were associated with any at-risk gambling (no difference between low-risk and moderate-risk/problem gambling) but attenuated after adjustment and only remained strong when comparing any at-risk gambling with no gambling at all.

**Table 4:** Proportion in the different categories for all available data and imputed data

All available data	Proportion	SE	95% CI	
Non-gamblers	0.318	0.008	0.304	0.333
Non-problem gamblers	0.485	0.008	0.470	0.501
Low-risk gamblers	0.152	0.006	0.141	0.164
Moderate-risk/problem gamblers	0.044	0.003	0.038	0.051
Imputed data	Proportion	SE	95% CI	
Non-gamblers	0.287	0.007	0.274	0.301
Non-problem gamblers	0.507	0.008	0.491	0.522
Low-risk gamblers	0.159	0.006	0.148	0.171
Moderate-risk/problem gamblers	0.046	0.003	0.040	0.053

**Table 5:** Multivariable analyses using imputed dataset of associations between PGSI categories at age 20 years and outcomes at age 24 years

	Non-gambler vs. non-problem		Non-gambler vs. low-risk		Non-gambler vs. moderate/problem		Non-problem vs. low-risk		Non-problem vs. moderate/problem		Low-risk vs. moderate/problem	
Mental health	OR	95% CI	OR	95% CI	OR	95% CI	OR	95% CI	OR	95% CI	OR	95% CI
Depression												
Unadjusted	*0.62*	*0.45, 0.84*	1.01	0.69, 1.46	1.31	0.73, 2.37	*1.63*	*1.14, 2.34*	*2.13*	*1.19, 3.82*	1.31	0.71, 2.41
Minimal model	*0.65*	*0.47, 0.91*	1.01	0.66, 1.54	1.37	0.70, 2.70	*1.55*	*1.05, 2.30*	*2.11*	*1.10, 4.03*	1.36	0.69, 2.67
Anxiety												
Unadjusted	*0.65*	*0.48, 0.87*	0.78	0.52, 1.17	0.84	0.41, 1.70	1.20	0.81, 1.76	1.29	0.64, 2.57	1.07	0.50, 2.29
Minimal model	*0.69*	*0.50, 0.94*	0.80	0.51, 1.24	0.84	0.38, 1.87	1.16	0.77, 1.76	1.22	0.56, 2.67	1.05	0.47, 2.37
Self-harm												
Unadjusted	0.83	0.67, 1.04	0.85	0.63, 1.16	0.70	0.41, 1.21	1.02	0.78, 1.34	0.84	0.49, 1.45	0.83	0.46, 1.47
Minimal model	0.84	0.66, 1.06	0.86	0.61, 1.21	0.70	0.38, 1.26	1.03	0.76, 1.39	0.83	0.46, 1.51	0.81	0.43, 1.53
Drugs & alcohol												
Illicit drugs												
Unadjusted	*1.34*	*1.12, 1.60*	*1.85*	*1.44, 2.39*	*2.61*	*1.72, 3.96*	*1.39*	*1.11, 1.74*	*1.95*	*1.29, 2.94*	1.41	0.89, 2.22
Minimal model	1.19	0.97, 1.47	*1.57*	*1.15, 2.14*	*1.90*	*1.16, 3.10*	*1.32*	*1.01, 1.71*	1.60	0.99, 2.56	1.21	0.72, 2.02
Smoking												
Unadjusted	*1.60*	*1.21, 2.12*	*2.54*	*1.75, 3.69*	*3.35*	*2.06, 5.43*	*1.59*	*1.17, 2.16*	*2.09*	*1.34, 3.25*	1.32	0.81, 2.14
Minimal model	1.29	0.93, 1.78	*1.96*	*1.26, 3.03*	*2.19*	*1.21, 3.96*	*1.52*	*1.06, 2.18*	1.70	0.98, 2.94	1.12	0.61, 2.04
Alcohol usage												
Mild												
Unadjusted	1.33	0.97, 1.84	*1.77*	*1.16, 2.69*	*3.99*	*2.22, 7.18*	1.32	0.89, 1.96	*2.99*	*1.68, 3.34*	*2.26*	*1.21, 4.23*
Minimal model	1.14	0.82, 1.58	1.27	0.82, 1.96	*2.53*	*1.32, 4.85*	1.11	0.74, 4.19	*2.23*	*1.19, 4.19*	*2.00*	*1.02, 3.94*
Moderate/severe												
Unadjusted	1.31	0.79, 2.17	1.54	0.78, 3.05	*7.70*	*3.88, 15.27*	1.17	0.63, 2.19	*5.87*	*3.14, 10.99*	*5.00*	*2.30, 10.86*
Minimal model	1.08	0.64, 1.83	1.03	0.50, 2.10	*4.35*	*2.02, 9.37*	0.95	0.50, 1.81	*4.04*	*2.01, 8.10*	*4.24*	*1.86, 9.67*
Social												
Crime												
Unadjusted	1.10	0.82, 1.49	*1.58*	*1.09, 2.27*	*2.35*	*1.40, 3.92*	*1.43*	*1.00, 2.03*	*2.12*	*1.32, 3.41*	1.49	0.88, 2.51
Minimal model	0.92	0.67, 1.26	1.14	0.77, 1.69	1.44	0.82, 2.52	1.24	0.86, 1.80	1.56	0.93, 2.64	1.26	0.72, 2.19
Unemployed												
Unadjusted	*0.58*	*0.47, 0.72*	*0.64*	*0.45, 0.90*	0.66	0.38, 1.15	1.09	0.78, 1.52	1.13	0.65, 1.96	1.04	0.57, 1.88
Minimal model	*0.56*	*0.45, 0.69*	*0.59*	*0.41, 0.83*	0.59	0.34, 1.04	1.05	0.75, 1.47	1.06	0.61, 1.84	1.01	0.56, 1.85
Social media use												
Unadjusted	*1.62*	*1.21, 2.19*	*2.03*	*1.33, 3.09*	*3.27*	*1.38, 7.74*	1.25	0.82, 1.91	2.02	0.86, 4.71	1.61	0.65, 3.98
Minimal model	*1.65*	*1.22, 2.23*	*2.36*	*1.54, 3.63*	*4.43*	*1.87, 10.52*	1.43	0.93, 2.21	*2.69*	*1.14, 6.32*	1.88	0.76, 4.64

Unadjusted and fully adjusted minimal model (significant (*p* < 0.05) covariates only; see Supplementary Table 5). Odds ratios shown are shown for all six pairwise comparisons. Highlighted in italics are significant at *p* < 0.05

Regular alcohol use was associated with any at-risk gambling, but a stronger association was seen with moderate-risk/problem gambling which remained after adjustment. More problematic use of alcohol was only associated with moderate-risk/problem gambling and remained very strongly associated even after adjustment. Involvement in crime was also higher in low-risk and moderate-risk/problem gamblers but associations attenuated after adjustment. Although any gambling at age 20 years was associated with high social media use at 24 years, odds ratios were highest in the moderate-risk/problem gambling group. Although no financial data were available on the economic consequences of problem gambling, non-problem and low-risk gamblers were more likely to be employed compared with non-gamblers.

Supplementary Table 5 summarizes the effect of covariates on all outcomes. The two covariates which influenced most of the outcomes were gender and regular smoking aged 16 years. Males were more likely to consume alcohol regularly and be involved in crime but less likely than females to be depressed, anxious, self-harming, and frequent users of social media. Apart from affecting smoking and alcohol use at 24 years, regular smokers at 16 years were also more likely to be anxious, self-harming, and involved with drugs and crime. Despite some strong effects of covariates, however, many of the individual associations with gambling on outcomes remained after adjustment. Adjustment for family factors had minimal effect on outcomes.

## Discussion

The ALSPAC Gambling Study, utilizing an existing cohort of young people with a wealth of background information, demonstrated that although many gambled without evidence of harm, a significant minority of this population sample of young people (5–7%) were classified as ‘moderate-risk/problem gambling’. Overall rates of moderate-risk and problem gambling remained stable between 20 and 24 years and those who were problem gamblers at age 20 were much more likely to remain problem gamblers at 24 years compared with those that were either low-risk or non-problem gamblers at 20 years. Our first hypothesis, that rates of problem gambling would remain stable, was supported. This study provides further evidence that problem gambling behaviours develop during late adolescence, with increased access to legal gambling after 18 years, and become established by the age of 20 years.

The frequency of any self-reported problems with gambling at age 20 and 24 years found in the ALSPAC (22–29%) is higher than the 10% reported in the Health Survey for England 2018 (NHS Digital (2019), and that found in other European countries (Kristiansen and Jensen 2014; Calado et al. 2017). Online betting and gambling were the most frequent gambling activities reported by moderate-risk/problem gamblers, consistent with other studies showing that young problem gamblers are more likely to gamble on the internet (Killick and Griffiths 2019), and evidence that online sports betting is being marketed at young men (Lopez-Gonzalez et al. 2017).

Moderate-risk and problem gamblers were predominantly males who were gambling every week, and previously had showed higher hyperactivity scores and conduct problems at 16 years, higher sensation seeking scores at 17 years, and were more external in their locus of control. Individual factors found to be associated with problem gambling at 20 years were largely consistent with the literature (Dowling et al. 2017), with recognized correlations with hyperactivity and impulsivity (Breyer et al. 2009,), and sensation seeking (Nower et al. 2004). An external locus of control has been associated with other potentially addictive behaviours, including the playing of video games (Lloyd et al. 2019). Problem gamblers were also more likely to have mothers who had problems with gambling, supporting the second hypothesis. Other studies (e.g. Griffiths et al. 2009) have consistently indicated that children of problem gamblers are two to four times more likely to develop gambling problems themselves than the children of non-problem gamblers. Dowling et al. (2018) suggested that gambling expectancies and motives were important pathways to the development of problem gambling in the offspring of problem gambling parents.

Problem gambling in the ALSPAC was associated with depression and other addictions, confirming the third hypothesis. The ALSPAC findings suggest a complex relationship between gambling and both anxiety and depression. Depression diagnosed at age 17 years was weakly associated with problem gambling at 20 years, and both low-risk and moderate-risk/problem gamblers were more likely to be depressed at age 24 years than non-problem gamblers. However, those that gambled without problems were less likely to be anxious or depressed compared with non-gamblers. This may be because the non-gamblers had a higher proportion of females who generally have higher rates of anxiety and depression at this age than males. Overall, it was not possible to conclude any causal direction of the relationship between depression and gambling, which may be reciprocally associated, as has been suggested by Dussault et al., (2011). Alternatively, the co-occurrence of gambling and depression in young adults may be better explained by a common underlying factor such as substance abuse (Chinneck et al. 2016).

Moderate-risk/problem gambling at 20 years was associated with regular smoking, problematic abuse of alcohol, and illicit drug use at 24 years, consistent with previous literature. A meta-analysis (Lorains et al. 2011) found that the highest mean prevalence of co-morbidities was for nicotine dependence (60.1%), followed by a substance use disorder (57.5%), any type of mood disorder (37.9%), and any type of anxiety disorder (37.4%). These correlates have been shown in many other studies of youth and young adult gambling. For example, a study of youth gambling in Norway also showed that male gender, depression, alcohol abuse, and dissociation were related to problem gambling (Molde et al. 2009). Young problem gamblers exhibit coping styles that are more emotion-based, avoidant, and distraction-oriented, and are more likely to engage in other addictive behaviours (Gupta et al. 2004). At-risk/problem internet gambling in young people has been shown to be associated with heavy alcohol use, low peer involvement, and poor academic functioning (Potenza et al. 2011). Longitudinal analysis of data from the Manitoba Longitudinal Study of Young Adults showed that at-risk or problem gambling for those aged 18–20 years was associated with major depressive disorder, alcohol dependence, and illegal drug use over a 5-year follow-up period (Afifi et al. 2016). Only illegal drug use at 18 years was predictive of at-risk or problem gambling during follow-up. Mutti-Packer et al. (2017) found a lack of mutual influence in problem gambling and alcohol misuse among adolescents and concluded that these behaviours have underlying risk factors in common, supporting the notion of a syndrome model of addiction.

The strength of the present study was its use of the large ALSPAC cohort, which has collected a wealth of data for over 25 years. When this cohort was initiated in 1991, it was representative of a whole community and it covered a range of environments from inner city to semi-rural in one geographical area. The ALSPAC has also collected a diverse range of psychological and physical measures from both the children and their families. Parental gambling activity was collected by self-report independently from their children’s report. Gambling activity at 20 and 24 years was self-reported by the young people, not by their parents, and detailed background information was available on these families. The novel findings of this study are (i) over 20% of young adult gamblers reported some problems associated with gambling, 5–7% were moderate-risk/problem gamblers, and the frequency of problem gambling remained stable between 20 and 24 years; (ii) problem gamblers were more likely to show characteristics of impulsivity, sensation seeking, and an external locus of control; and (iii) there were strong associations of moderate-risk/problem gambling with nicotine dependence and problematic use of alcohol and illicit drugs at the age of 24 years.

The main limitation of this research is the missing data, with less than 50% returning the survey at age 20 years, and not all gamblers completed the PGSI. Multiple imputation techniques were used to minimize the bias from attrition, but the analyses probably underestimated the prevalence of problem gambling. This was compounded by a significant gender bias, with the final sample comprising more females than males, whereas males were more likely to be at risk of problem gambling. However, it is worth noting that the univariate odds ratios using all available data and using imputed data were very similar.

## Conclusions

A small but significant minority of young people (mainly males) show problem gambling behaviours which are associated with other potentially harmful addictions. Problematic gambling behaviours appear to be established by the age of 20 years and they remained stable when measured 4 years later. The implications of this study are that educational and legislative interventions to prevent problems with gambling need to target adolescents and emerging adults, and that treatment interventions for young adult problem gamblers should include support for mental health, as well as abuse of alcohol and drugs.

## Electronic Supplementary Material

ESM 1 (DOCX 59 kb)
