# Supplementary material for: Problem Gambling in Early Adulthood: a Population-Based Study
Source: Int J Ment Health Addict. 2020 Oct 26;20(2):754–70. doi: 10.1007/s11469-020-00401-1 (PMC8930883; doi:10.1007/s11469-020-00401-1)
Supplement: Supplementary file 1 — (DOCX 59 kb) [file 11469_2020_401_MOESM1_ESM.docx]

**Supplementary Material 1. Missing data and multiple imputation.**

Missing data is a widespread problem in longitudinal cohort studies such as ALSPAC. At age 20, only 48% of participants returned the questionnaire (online 63%, paper 37%). Even amongst those that answered the questionnaires, up to 43% had missing data on either outcomes or antecedents (see below table). Furthermore, 414 participants at age 20 answered that they had participated in some form of gambling in the past 12 months but did not answer the follow up problem gambling (PGSI) questions. Of these, 87% were less than weekly gamblers and 76% were paper questionnaires. They were more likely to be females, have lower IQ, more external locus of control and from mothers with low educational background (data available on request). It is likely that without taking this into account, our results would be biased. Multiple imputation is a common technique used to correct for bias introduced by loss to follow-up (Sterne et al. 2009). We used multiple imputation by chained equations using the “mi impute” command in Stata v.15.1 (StataCorp. 2017). We imputed up to the number of participants where gambling status (yes/no) could be established (N = 4263) using 50 imputations. Imputation model diagnostics were performed using the command ‘midiagplots’ which compares the distributions of the observed, imputed, and completed values (Eddings & Marchenko 2012). Binary variables and categorical variables used logistic, ordinal and multinomial regression, as appropriate. Normally distributed variables were incorporated using linear regression in the imputation model. PGSI was imputed conditional on gambling status, i.e. only those who did gamble were imputed for this variable. A number of auxiliary variables, associated with missingness, were included in the final imputation model, together with those included in the final regression models.

References

Sterne JA, White IR, Carlin JB, Spratt M, Royston P, Kenward MG, Wood AM, Carpenter JR. 2009. Multiple imputation for missing data in epidemiological and clinical research: potential and pitfalls. *BMJ*,*38,*b2393.

Eddings W, Marchenko Y. 2012. Diagnostics for multiple imputation in Stata. *Stata Journal*, *12*(3),353

Table of imputed % for all variables used in analyses

| **Variable** | **Complete** | **Imputed** | **Imputed %** |
| --- | --- | --- | --- |
| Gender | 4263 | 0 | 0.0% |
| Maternal education | 3971 | 292 | 6.8% |
| Financial difficulties in pregnancy | 3879 | 384 | 9.0% |
| PGSI at 20 | 2596 | 414 | 13.8% |
| IQ at 8 | 3319 | 944 | 22.1% |
| Smoking at 16.5 | 3260 | 1003 | 23.5% |
| Hyperactivity at 16.5 | 3174 | 1089 | 25.5% |
| Conduct problems at 16.5 | 3170 | 1093 | 25.6% |
| Video games at 13 | 3159 | 1104 | 25.9% |
| Locus of control at 16.5 | 3069 | 1194 | 28.0% |
| Alcohol use at 16.5 | 3033 | 1230 | 28.9% |
| Social media at 24 | 2849 | 1414 | 33.2% |
| Mental well-being at 17.5 | 2794 | 1469 | 34.5% |
| Depression at 17 | 2767 | 1496 | 35.1% |
| Parental monitoring | 2675 | 1588 | 37.3% |
| Illicit drugs at 24 | 2582 | 1681 | 39.4% |
| Self-harm at 24 | 2577 | 1686 | 39.5% |
| Depression at 24 | 2576 | 1687 | 39.6% |
| Smoking at 24 | 2572 | 1691 | 39.7% |
| Anxiety at 24 | 2571 | 1692 | 39.7% |
| Crime at 24 | 2567 | 1696 | 39.8% |
| Employment at 24 | 2530 | 1733 | 40.7% |
| Maternal problem gambling | 2511 | 1752 | 41.1% |
| Alcohol use at 24 | 2457 | 1806 | 42.4% |
| Sensation seeking at 17 | 2430 | 1833 | 43.0% |

**Supplementary Table 1. Gambling activities included in the 20-year survey**. **Derived from the British Gambling Prevalence Survey.**

| Activity | Include | Does not include |
| --- | --- | --- |
| 1.Lottery games | Lotto, Thunderball and Euromillions | Scratchcards |
| 2.Scratchcards | Lottery scratchcard games played offline and online | Newspaper or magazine scratchcards |
| 3.Any other lottery tickets | Charity lotteries for hospices, sports or social clubs, e.g. "Monday Lottery" | Irish Lottery or any other international lotteries or buying raffle tickets |
| 4.Football pools | - | Betting on football matches with a bookmaker |
| 5.Bingo cards or tickets | Playing boards at a Bingo Hall | Newspaper bingo tickets, or bingo played on-line |
| 6.Fruit (slot) machines | - | Quiz machines |
| 7.Virtual gaming machines | Betting on virtual roulette, keno, bingo etc in a bookmaker’s | Quiz machines |
| 8.Table games | Roulette, dice or cards in a casino | Poker or casino games played online |
| 9.Online gambling | Playing poker, bingo, slot machine style games, or casino games for money online through a computer, mobile phone or interactive TV | Bets made with online bookmakers or betting exchanges |
| 10.Online betting with a bookmaker | Betting online through a computer, mobile phone or interactive TV on any event or sport | Bets made with a betting exchange or spread betting |
| 11.Betting exchange | Peer to peer betting | - |
| 12.Betting on horse races | Betting on horse races in a bookmaker’s, by phone, or at the track. Also includes tote betting and betting on virtual horse races shown in a bookmaker’s | Bets made with on-line bookmakers or betting exchanges |
| 13.Betting on other event or sport. | Betting on any other event or sport at the bookmakers, by phone or at the venue. Also includes Irish Lottery, 49s | Bets made with online bookmakers or betting exchanges or spread betting |
| 14.Spread-betting | - | - |
| 15.Private betting | Playing cards or games for money with friends, family or colleagues | - |
| 16.Any other gambling form | - | - |

**Supplementary Table 2. Parental monitoring items answered at age 17.**

| *Parental monitoring items* |
| --- |
| 1. How often do your carers / parents know what you do during your free time? |
| 1. How often do your carers / parents know what you spend your money on? |
| 1. How often, during the last month, have your carers / parents been unaware of where you were at night? |
| 1. How often do you keep secrets from your carers / parents about what you do during your free time? |
| 1. How often do you keep things from your carers / parents about what you do during nights and weekends? |
| 1. How often do you tell your carers / parents about what you did and where you went during the evening? |
| 1. How often do your carers / parents ask you about what happened during your free time? |
| 1. How often were conversations about your spare time started by your carers / parents during the past month? |
| 1. How often do your carers / parents take the time to listen to you when you talk about what happened during your free time? |
| 1. How often do you have to have your carers / parents’ permission before you go out on weeknights? |
| 1. How often do your carers / parents demand to know where you are in the evenings, who you are going to be with and what you are going to do before you go out? |
| 1. How often do your carers / parents ask you to tell them how you spend your money? |
|  |
| Response options for each question were: ‘never’, ‘hardly ever’, ‘sometimes’, ‘most of the time’, and ‘always’. Items were reverse coded, if necessary, so that higher scores reflected a greater degree of parental monitoring. |

**Supplementary Table 3. Variables used in analyses.**

| Variable | Age (years) | Description |
| --- | --- | --- |
| *Child antecedents* |  |  |
| Gender | Birth |  |
| IQ | 8 | A short version of the WISC III^1^ applied by trained psychologists in research clinic was used. We used the total IQ (verbal + performance) and compared the % of people in the bottom quartile to the rest. |
| Video games | 13 | Teenagers were asked whether they chose to play computer games with other children instead of other activities. This was used as a binary yes/no variable. |
| Hyperactivity and conduct problems | 16.5 | Measured using the Strengths and Difficulties Questionnaire (SDQ). Scores were entered as binary variables based on cut-offs for ‘abnormal’ scoring on each SDQ subscale as suggested by Goodman.^2^ |
| Locus of control | 16.5 | Calculated summing the answers on a 12 item Nowicki-Strickland Locus Of Control Scale^3^. People with a lower score believe that an outcome is largely contingent upon their own behaviour and are having a more *internal* locus of control, whereas those with a higher score believe that luck, fate, chance or powerful others largely determine an outcome are more *external*. Scores greater than the median were labelled external and less than or equal to the median were labelled internal. |
| Sensation seeking | 17 | A total sensation seeking score (novelty subscale + intensity subscale) was measured using the Arnett Sensation Seeking Scale^4^. A higher score indicates a higher tendency to pursue sensory pleasure and excitement. |
| Mental well being | 17.5 | The Warwick-Edinburgh Mental Well-being Scale (WEMWBS) is a 14-item scale of mental well-being covering subjective well-being and psychological functioning, in which all items are worded positively, and address aspects of positive mental health. The scale is scored by summing responses to 14, each item answered on a 1 to 5 Likert scale. The minimum scale score is 14 and the maximum is 70. |
| Depression | 17 | We used an ICD-10 diagnosis of depression (yes/no) established in a research clinic at age 17. |
| Smoking | 16.5 | At age 16.5 participants were asked about cigarette smoking habits. We used the % of weekly smokers compared to those that did not smoke weekly. |
| Alcohol use | 16.5 | At age 16.5 participants were asked about alcohol use habits. We used the % of weekly consumers compared to those that did not consume alcohol weekly. |
| Education/employment status | 17 | Participants were asked whether they were in education or employment (full or part-time). This was used as a binary yes/no variable. |
| Regular gambling | 20 | Those that gamble weekly were compared to those that gambled less than weekly. |
| *Maternal/socioeconomic antecedents* |  |  |
| Maternal education | 32 weeks gest. | Measured as the highest education level the mother held. It was classified as CSE (Certificate of Secondary Education)/none, Vocational, O level, A level, Degree. We compared the proportion of mothers with a degree compared to those with levels below a degree. |
| Maternal problem gambling | Child aged 18 | Collected using the Canadian Problem Gambling Index^6^ where mothers were classified into non-gamblers, no-problem gamblers, low risk gamblers, moderate risk gamblers and problem gamblers. |
| Financial difficulties | 32 weeks gest. | A numerical score was created from five questions about how difficult the mothers found affording certain items. The higher the score the more financial difficulties. We compared the top tertile with the rest. |
| Parental monitoring | 17 | Information on parental monitoring was provided by the young person (completed independently of their parents) at the same age as the first gambling questionnaire using a computerized 12‐item self‐report (see supplementary material Table 2 for a list of items). |
| *Outcomes* |  |  |
| Depression | 24 | The Computerised Interview Schedule – Revised (CIS-R) is a self-administered computerized interview which derives diagnoses based on ICD-10 criteria for depression and anxiety disorder (yes/no).^7^ |
| Anxiety | 24 | The Computerised Interview Schedule – Revised (CIS-R) is a self-administered computerized interview which derives diagnoses based on ICD-10 criteria for depression and anxiety disorder (yes/no)^7^ |
| Self-harm | 24 | Ever attempted self-harm (yes/no). Part of the CIS-R (see above). |
| Crime | 24 | Whether participant has ever engaged in violent (includes snatching with force, fighting and carrying a weapon) or non-violent crime (includes shoplifting, vandalism, breaking in vehicle, joyriding, selling drugs, breaking into house, selling stolen goods, arson, snatching without force, buying stolen goods, fraud, and claiming untitled benefits) in the past 12 months (yes/no).^8^ |
| Illicit drugs | 24 | Whether participant has used drugs such as cocaine, crack, ecstasy etc. in the past 12 months (yes/no). |
| Smoking cigarettes | 24 | Frequency of smoking cigarettes. We compared weekly or more smoking to the rest. |
| Alcohol consumption | 24 | We used ‘alcohol use disorder scores’ as defined by the Diagnostic and Statistical Manual of Mental Disorders V (DSM-V)^9^. We compared those that scored for moderate/severe disorder to those that scored for mild and none. |
| Employment status | 24 | Whether participant is in part-time or full employment. |
| Social media use | 24 | Measured as the frequency of using social media. We compared the % using it >10 times/day to those that used it less frequently. |

# *References*

1. Wechsler D, Golombok S, Rust J. 1992. *WISC-III UK: Wechsler Intelligence Scale for Children.* Sidcup, UK: Psychological Corporation.
2. Goodman A, Goodman R. 2009. Strengths and difficulties questionnaire as a dimensional measure of child mental health. *Journal of the American Academy of Child and Adolescent Psychiatr,* *48*,400-403.
3. Nowicki, S. & Strickland, BR. 1973. A locus of control scale for children. *Journal of Consulting and Clinical Psychology, 40,* 148-154.
4. Arnett, J. 1994. Sensation Seeking: A new conceptualization and a new scale. *Personality and Individual Differences,16****,***289-286
5. Stewart-Brown, S., & Janmohamed, K. (2008). Warwick-Edinburgh mental well-being scale. *User guide. Version*, *1*.
6. Ferris, J. & Wynne, H. 2001. The Canadian problem gambling index: Final report. Submitted for the Canadian Centre on Substance Abuse.
7. Patton G, Coffey C, Posterino M, Carlin J, Wolfe R, Bowes G. 1999. A computerised screening instrument for adolescent depression: population-based validation and application to a two-phase case-control study. *Social Psychiatry and Psychiatric Epidemiology*, *34*,166–172
8. Smith DJ, McVie S, Woodward R, Shute J, Flint J, McAra L. 2001. *The Edinburgh study of youth transitions and crime: Key findings at ages 12 and 13*. Edinburgh Study of Youth Transitions and Crime Research Digest No. 1.
9. Regier, D. A., Narrow, W. E., Kuhl, E. A., & Kupfer, D. J. (2009). The conceptual development of DSM-V. *American Journal of Psychiatry*, *166*(6), 645-650.

**Supplementary table 4.** Comparing univariate odds ratios for imputed data and all available data.

|  | **Imputed (N=4263)** | **All available** |
| --- | --- | --- |
|  | **Unadjusted OR (95% CI)** | **Unadjusted OR (95% CI)** |
| *Mental health* |  |  |
| **Depression at 24**  Non-gambler (*Ref)*  Non-problem gambling  Low risk gambling  Moderate/problem | **0.62 (0.45, 0.84)**  1.01 (0.69, 1.46)  1.31 (0.73, 2.37) | *N=2353*  **0.61 (0.45, 0.83)**  1.00 (0.67, 1.50)  1.22 (0.65, 2.28) |
| **Anxiety at 24**  Non-gambler (*Ref)*  Non-problem gambling  Low risk gambling  Moderate/problem | **0.65 (0.48, 0.87)**  0.78 (0.52, 1.17)  0.84 (0.41, 1.70) | *N=2348*  **0.64 (0.47, 0.87)**  0.75 (0.48, 1.17)  0.75 (0.35, 1.61) |
| **Self-harm at 24**  Non-gambler (*Ref)*  Non-problem gambling  Low risk gambling  Moderate/problem | 0.83 (0.67, 1.04)  0.85 (0.63, 1.16)  0.70 (0.41, 1.21) | *N=2354*  0.91 (0.73, 1.13)  0.91 (0.66, 1.24)  0.72 (0.41, 1.27) |
|  |  |  |
| *Drugs & alcohol* |  |  |
| **Illicit drugs at 24**  Non-gambler (*Ref)*  Non-problem gambling  Low risk gambling  Moderate/problem | **1.34 (1.12, 1.60)**  **1.85 (1.44, 2.39)**  **2.61 (1.72, 3.96)** | *N=2359*  **1.34 (1.12, 1.61)**  **1.80 (1.39, 2.34)**  **2.79 (1.76, 4.42)** |
| **Smoking weekly at 24**  Non-gambler (*Ref)*  Non-problem gambling  Low risk gambling  Moderate/problem | **1.60 (1.21, 2.12)**  **2.54 (1.75, 3.69)**  **3.35 (2.06, 5.43)** | *N=2351*  **1.64 (1.23, 2.19)**  **2.29 (1.59, 3.29)**  **3.49 (2.08, 5.87)** |
| **Alcohol disorder at 24**  **Mild**  Non-gambler (*Ref)*  Non-problem gambling  Low risk gambling  Moderate/problem  **Moderate/severe**  Non-gambler (*Ref)*  Non-problem gambling  Low risk gambling  Moderate/problem | 1.33 (0.97, 1.84)  **1.77 (1.16, 2.69)**  **3.99 (2.22, 7.18)**  1.31 (0.79, 2.17)  1.54 (0.78. 3.05)  **7.70 (3.88, 15.27)** | *N=2252*  **1.49 (1.03, 2.17)**  **1.87 (1.16, 3.02)**  **4.50 (2.38, 8.49)**  1.38 (0.80, 2.38)  1.01 (0.44, 2.31)  **9.67 (4.75, 19.70)** |
|  |  |  |
| *Social* |  |  |
| **Crime at 24**  Non-gambler (*Ref)*  Non-problem gambling  Low risk gambling  Moderate/problem | 1.10 (0.82, 1.49)  **1.58 (1.09, 2.27)**  **2.35 (1.40, 3.92)** | *N=2347*  1.14 (0.85, 1.52)  **1.48 (1.01, 2.17)**  **2.62 (1.54, 4.46)** |
| **Unemployed at 24**  Non-gambler (*Ref)*  Non-problem gambling  Low risk gambling  Moderate/problem | **0.58 (0.47, 0.72)**  **0.64 (0.45, 0.90)**  0.66 (0.38, 1.15) | *N=2313*  **0.60 (0.48, 0.76)**  **0.65 (0.46, 0.91)**  0.67 (0.38, 1.18) |
| **Social media use at 24**  **>10 times/day**  Non-gambler (*Ref)*  Non-problem gambling  Low risk gambling  Moderate/problem | **1.62 (1.21, 2.19)**  **2.03 (1.35, 3.09)**  **3.27 (1.38, 7.74)** | *N=2589*  **1.83 (1.33, 2.51)**  **2.14 (1.34, 3.40)**  **2.96 (1.23, 7.10)** |

**Supplementary table 5.** Significant covariates remaining in minimal models are highlighted in grey. All odds ratios are above 1 except for gender effects on depression, anxiety, self-harm and social media use where males are less likely to be depressed, anxious, self-harming, and frequently use social media. Also, those in the lowest 25% of well-being were less likely to be a frequent social media user.

|  | **Depression** | **Anxiety** | **Self-harm** | **Illicit drugs** | **Smoking** | **Alcohol** | | **Crime** | **Unemploy-ment** | **Social media use** |
| --- | --- | --- | --- | --- | --- | --- | --- | --- | --- | --- |
| *Covariates* |  |  |  |  |  | **Mild** | **Moderate/severe** |  |  |  |
| Gender (male) |  |  |  |  |  |  |  |  |  |  |
| IQ at 8 (lowest 25%) |  |  |  |  |  |  |  |  |  |  |
| Video games (yes) |  |  |  |  |  |  |  |  |  |  |
| Hyperactivity (abnormal) |  |  |  |  |  |  |  |  |  |  |
| Conduct problems (abnormal) |  |  |  |  |  |  |  |  |  |  |
| Locus of control (>median) |  |  |  |  |  |  |  |  |  |  |
| Sensation seeking (continuous increasing scale) |  |  |  |  |  |  |  |  |  |  |
| Mental well-being  (lowest 25%) |  |  |  |  |  |  |  |  |  |  |
| Depression (yes) |  |  |  |  |  |  |  |  |  |  |
| Education/employment (no) |  |  |  |  |  |  |  |  |  |  |
| Smoking at 16 (% weekly) |  |  |  |  |  |  |  |  |  |  |
| Alcohol use at 16 (% weekly) |  |  |  |  |  |  |  |  |  |  |
| Maternal education  (>A level) |  |  |  |  |  |  |  |  |  |  |
| Maternal financial difficulties (high score) |  |  |  |  |  |  |  |  |  |  |
| Maternal PGSI  (at risk/problem) |  |  |  |  |  |  |  |  |  |  |
| Parental monitoring (lowest 25%) |  |  |  |  |  |  |  |  |  |  |
